# Analysis of Global Gene Expression in Maize (*Zea mays*) Vegetative and Reproductive Tissues That Differ in Accumulation of Starch and Sucrose

**DOI:** 10.3390/plants11030238

**Published:** 2022-01-18

**Authors:** Cristal López-González, Sheila Juárez-Colunga, Samuel Trachsel, Nayelli Marsch-Martínez, C. Stewart Gillmor, Axel Tiessen

**Affiliations:** 1 Departamento de Ingeniería Genética, Unidad Irapuato, CINVESTAV-IPN, Irapuato 36824, Mexico; shejuacol@gmail.com; 2 Global Maize Program, International Maize and Wheat Improvement Center (CIMMYT), El Batán, Texcoco 56237, Mexico; tracselsam@gmail.com; 3 Departamento de Biotecnología y Bioquímica, Unidad Irapuato, CINVESTAV-IPN, Irapuato 36824, Mexico; nayelli.marsch@cinvestav.mx; 4 Langebio, Unidad de Genómica Avanzada, CINVESTAV-IPN, Irapuato 36824, Mexico; stewart.gillmor@cinvestav.mx

**Keywords:** *Zea mays*, maize stem, female inflorescence, transcriptome analysis, sucrose–starch metabolism

## Abstract

Carbon allocation between vegetative and reproductive tissues impacts cereal grain production. Despite great agricultural importance, sink–source relationships have not been fully characterized at the early reproductive stages in maize. Here, we quantify the accumulation of non-structural carbohydrates and patterns of gene expression in the top internode of the stem and the female inflorescence of maize at the onset of grain filling (reproductive stage R1). Top internode stem and female inflorescence tissues of the Puma maize inbred line were collected at reproductive stage R1 (without pollination) and non-structural carbohydrates were quantified by spectrophotometry. The female inflorescence accumulated starch at higher levels than the top internode of the stem. Global mRNA transcript levels were then evaluated in both tissues by RNA sequencing. Gene expression analysis identified 491 genes differentially expressed between the female inflorescence and the top stem internode. Gene ontology classification of differentially expressed genes showed enrichment for sucrose synthesis, the light-dependent reactions of photosynthesis, and transmembrane transporters. Our results suggest that sugar transporters play a key role in sugar partitioning in the maize stem and reveal previously uncharacterized differences between the female inflorescence and the top internode of the stem at early reproductive stages.

## 1. Introduction

Yield and harvest indices in cereals are impacted by the partitioning of carbon between vegetative and reproductive tissues. Photoassimilates produced in the source tissues travel through the phloem as sucrose and accumulate in sink tissues as starch [1,2,3]. Comparative analysis of different maize varieties has shown that the strength of the sink–source relationship is established at the early stages of grain filling and has a significant impact on the final kernel weight [4].

Source strength is determined by both the rate of photosynthesis and the rate of photoassimilate export from source tissues [3,5]. Photoassimilate is primarily transported in the form of sucrose. Triose phosphates produced in the chloroplast by photosynthesis are shuttled to the cytosol and are used to synthesize sucrose through the sequential action of fructose 1, 6-biphosphatase (F16BP), sucrose phosphate synthase (SPS), and sucrose phosphate phosphatase (SPP) [6,7]. Long distance sucrose transport occurs through the phloem [5] via the SUT and SWEET sucrose transporter families. In rice, increasing sucrose loading into the phloem through the expression of an *Arabidopsis* sucrose transporter (*SUC2*) has been reported to increase grain yield by 16% relative to control plants [6]. Once sucrose reaches a sink tissue, it is unloaded and can be broken down either by sucrose synthase (SUS) into fructose and UDP-glucose or by invertase (INV) into fructose and sucrose. A simplified measure of sink strength can be obtained as the sink size (total biomass of sink tissue in g) per unit of sink activity (specific rate of resource uptake in mol g^−1^ s^−1^) [2,7]. Sucrose degradation in the sink tissue will drive further sucrose import, and the levels of SUS and INV activity are a major determinant of sink strength [8,9,10]. In maize, increased SUS activity results in greater starch accumulation [11].

To ensure a consistent supply of carbon to support metabolism and growth, much of the photoassimilate delivered to a sink tissue will be stored as starch [8,9]. As such, starch plays a dual role in carbon allocation, acting as both a source, releasing carbon reserves in leaves for growth and development, and as a sink, either as a dedicated starch store (in seeds and tubers) or as a temporary reserve of carbon contributing to sink strength in organs such as flowers, fruits, and developing non-starchy seeds [8,10]. Starch synthesis is catalyzed by ADP-glucose pyrophosphorylase (AGPase), starch synthases (SS), starch branching and debranching enzymes (DBE and SBE), and the granule-bound starch synthase (GBSS) [10,12,13,14]. Overexpression and increasing the catalytic activity of AGPase subunits can promote a slight increase in crop yield [15,16,17,18]. Other studies have focused on transcription factors regulating starch metabolism enzymes in different crops [19]. Transcription factors such as ZmbZIP91, ZmEREB156, and OsbZIP58 have been found to be involved in the regulation of starch synthesis enzymes in the endosperm [20,21,22], while OsCRCT regulated starch synthesis in vegetative tissues [23]. This suggests that starch regulation mechanisms differ depending on the type of starch that is synthesized.

The transcriptional regulation of sugar partitioning and sink–source communication has not been widely studied [24]. Further study of sink–source relations and the transcriptional regulation of sucrose–starch metabolism in the stem and female inflorescence before pollination can point to important enzymes that can be considered to increase crop yield, such as the transporters involved in the reallocation and partitioning of carbon and their possible regulation mechanisms. To begin to address this deficit, we have characterized non-structural carbohydrate partitioning and transcript populations in stem and female inflorescence tissues at an early reproductive stage in maize. Our analysis indicates that sugar transport is highly active in the top internode section of the stem, while the female inflorescence is the main sink tissue at the early reproductive stage.

## 2. Materials and Methods

### 2.1. Plant Growth and Material

#### 2.1.1. Field Grown Maize for Metabolite Quantification

Two commercial maize hybrids, white grain Puma (Asgrow) and yellow grain Dow2B (Dow), were grown in the summer at the Experimental Field of the University of Guanajuato, in Irapuato, Guanajuato, Mexico. A standardized method with a mechanical juice extractor (International, model EXS) was used to extract juice from whole maize stems, including the leaf sheaths. Juice samples were obtained in triplicate. Samples were collected weekly, before, during, and after pollination, from 60 days after sowing (DAS) to 102 DAS. Stem juice aliquots of 1 mL were put in a 96-well microplate, immediately frozen on dry ice, and stored at −20 °C until metabolite quantification.

#### 2.1.2. Greenhouse Grown Maize for Carbohydrate Quantification and RNA Sequencing

Puma hybrid maize plants were grown in a greenhouse in Irapuato, Guanajuato, Mexico, in 10 L plastic pots containing 1/1 *v*/*v* peatmoss and a vermiculite sterile soil mixture with optimal watering and fertilization, under natural light with no supplemental light, during the months of May to August, when the daylength was from 13 to 14 h. Peak daylight intensity was approximately 250 µmol/m^2^/s, which was measured at the level of the plant female inflorescence, and the peak greenhouse temperature was 28 °C. Plant tissues were collected at early reproductive stage R1 (60 DAS) between 8 a.m. and 12 p.m. Tissues collected were root, RT; nodes, ND; the top internode section of the stem, TI; the bottom internode section of the stem, BI; leaf sheath, LS; leaf blade, LB; and female inflorescence (FI), which were bagged prior to silk emergence. Similar tissues (RT, ND, TI, BI, LS, and LB), with the exception of FI, were collected in Puma hybrid maize plants at 45 DAS (vegetative stage, v12). Samples were frozen, milled with a Retsch mill in liquid nitrogen, and stored at −80 °C until carbohydrate determination and RNA extraction were needed.

### 2.2. Iodine Staining

Puma maize stem tissues were collected at 21 DAS. Longitudinal and cross sections of maize stems that were 2–3 mm thick were stained with KI-I_2_ solution. Images of 21 DAS plants were taken with a Leica EZ4 (Leica Application Suite v3.4.0) stereo microscope.

### 2.3. Non-Structural Carbohydrate Quantification

Soluble sugars (glucose, Glc; fructose, Frc; sucrose, Suc) and starch were measured using an enzymatically coupled method as described by [25]. Stem juices (1 mL aliquots) were centrifuged at 4000 rpm (in a SORVALL RT7 Plus Centrifuge; Newtown, CT, USA), supernatants were collected in another deep-well microplate, and 10 μL aliquots of diluted samples were used for Frc, Glc, and Suc measurements. All samples were diluted between 1:50 and 1:200 *v*/*v*. For starch extraction, pellets were washed three times with 80% EtOH (*v*/*v*), and then soluble-sugar-free pellets were incubated with 500 μL 10 mM NaOH at 100 °C for 2.5 h. Starch was hydrolyzed in 50 mM Hepes (pH 7.5) at 37 °C overnight with the addition of 10 units of a-amylase (EC 3.2.1.1; Roche) and 10 units of amyloglucosidase (EC 3.2.1.3; Roche). The reaction mix was kept overnight at 37 °C to allow breakdown of the polymeric chains to glucose molecules. Finally, samples were centrifuged at 4000 rpm, and 10–20 µL were used for starch quantification. Puma hybrid samples at V12 and R1 stages were lyophilized, and carbohydrates were measured using a method described by [26], using the same reagents as indicated for the previous method.

### 2.4. RNA Extraction

Total RNA from female inflorescences (FI) and top internodes of the stem (TI) at 60 DAS were extracted using the PureLink RNA-micro-to-midi Kit (Invitrogen Corp., Carlsbad, CA, USA), as described by the manufacturer. The purity and integrity of the RNA samples were evaluated by electrophoresis on 2% RNase-free agarose gels, spectrophotometry (A260/A280 and A260/A230 ratios) using a NanoDrop 2000 (Thermo Fisher Scientific, Waltham, MA, USA), and quality analysis using the Agilent 2100 Bioanalyzer system (Agilent technologies, Inc, Santa Clara, CA, USA).

### 2.5. RNAseq Analysis

RNA sequencing libraries from two biological replicates each of FI and TI tissues were constructed according to the standard Illumina protocol and were sequenced using the Illumina MySeq™ 2000 platform to generate 2 × 300-nucleotide paired-end reads. This was performed as a service by the National Laboratory of Genomics for Biodiversity (Langebio) at Cinvestav, Irapuato, Mexico. RNA-seq read quality was assessed using FastQC v0.11.2 and was cleaned using the Trimmomatic software [27]. Reads were aligned to the maize B73 reference genome (ZmB73_RefGen_v4, www.maizegdb.org (accessed on 1 December 2020)) using TopHat v2.0.13 software. The expression level was calculated in fragments per kilobase of transcript per million fragments mapped (FPKM), as described previously [28,29]. Raw data have been deposited in the GEO database under accession number GSE181998.

### 2.6. Differential Gene Expression Analysis

Differentially expressed genes (DEGs) were identified through pairwise comparison using EdgeR software, and the *p*-values were adjusted using the Benjamini–Hochberg procedure to determine the false discovery rate (FDR) [30,31,32]. Only the genes with FPKM results that met the criteria of FDR < 0.05 and fold change > |2| between the two conditions were considered to be differentially expressed. Data analyses were updated using CLC Genomics Workbench 20.0 (QIAGEN) software mapping to the B73 RefGen_v4 (https://www.maizegdb.org/genome/assembly/Zm-B73-REFERENCE-GRAMENE-4.0 (accessed on 1 December 2020)).

### 2.7. GO Enrichment Analysis

The list of DEGs was analyzed for GO enrichment using the ClueGO and Cluepedia [33] plug-ins in Cytoscape v3.8.0. DEGs that presented increased expression in each tissue (FI, TI) were analyzed separately to determine the main GO-enriched term in each tissue. Another analysis was performed in the Panther database (available online: http://geneontology.org/ accessed on 3 November 2020) [34,35]. The GO categories considered for the analysis included cellular component, molecular function, and biological process. After the hypergeometric test, Bonferroni correction was employed for *p*-value correction, with a cut-off of 0.05. The GO terms satisfying the condition were considered to be significantly enriched in the DEGs list.

### 2.8. Quantitative Real-Time PCR (qRT-PCR)

Total RNA samples (2 µg) were reverse transcribed to generate the first strand cDNA using an oligo dT20 primer and 200 units of SuperScript II reverse transcriptase (Invitrogen). All primer design (Table S1) and qRT-PCR reactions were performed as described by [36]. Three biological replicates for each tissue were stored at −80 °C and were subjected to independent extraction procedures and qRT-PCR analysis on the same 96-well plate and on independent plates. qRT-PCR was performed with a CFX™ Real-Time PCR System (BioRad). A mastermix was made using 40 ng of reverse transcribed total RNA; 1.2 mM each of dNTP (dATP, dCTP, dGTP, dTTP); and SYBR Green TaqReadyMix™, 2X. The total reaction volume was 20 μL. The following PCR protocol was used: denaturation program (95 °C for 3 min) and amplification and quantification program repeated 40× (95 °C for 15 s, 60 °C for 30 s); a melting curve program was also used (60–95 °C, with a heating rate of 0.3 °C per second). To compare gene expression between tissues, we used the modified absolute gene expression data analysis method [37,38] described by [36,39]. qRT-PCR data were reported as number of molecules at cycle zero (*N*_0_) in *log*_10_ of the fluorescence units (RFU).

### 2.9. Gene Expression of Sucrose-Starch Genes in B73 Genome Atlas

Expression data for DEGs between the female inflorescence and the top internode and for the *SUT* and *SWEET* transporter families were extracted from the B73 genome atlas. Spearman correlation coefficients were calculated with the Hmisc and corrplot libraries in RStudio v1.1.423. Heatmaps were built with gplots and RColorBrewer libraries. A co-expression network built with the 75 tissue samples from the B73 genome atlas data was explored through the COBrowser website to search for transcription factors that were co-expressed with sucrose–starch genes (Schaefer et al., 2014; Stelpflug et al., 2016). Whole transcription factor and sucrose–starch genes were identified from Grassius (available online: https://grassius.org/, accessed on 1 December 2020) [40], the PlantPAN database (available online: http://plantpan.itps.ncku.edu.tw, accessed on 1 December 2020) and the MaizeGDB database (available online: https://www.maizegdb.org, accessed on 1 December 2020), respectively. Gene expression patterns in photosynthetic tissues were graphed with Excel software.

### 2.10. Statistical Analysis

All statistical analysis of carbohydrate measurements were performed using the Agricolae v1.3-2 package in RStudio v.1.1.423 (RStudio Team, 2020). Data were analyzed using an ANOVA with multiple comparison applying a Fisher–LDS test (*p*-value = 0.05) with Bonferroni correction. Averages ± SE and *t*-tests were calculated using Excel software.

## 3. Results

### 3.1. Hexose to Sucrose Ratio Increases at Early Reproductive Stages

**Figure 1:**
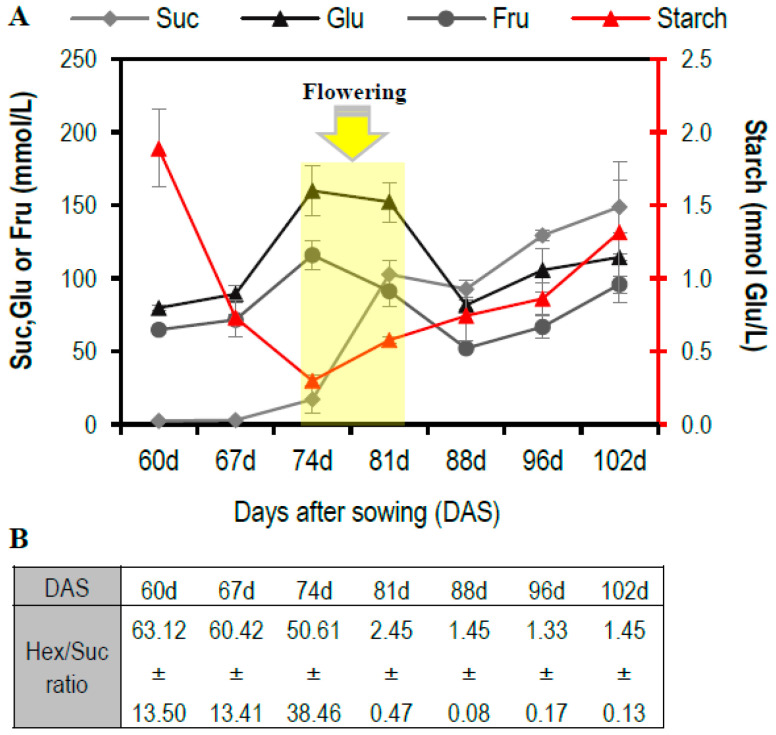
Carbohydrate accumulation in stem juice of the white grain Puma maize hybrid at the reproductive stage. (**A**) Non-structural carbohydrates in maize stem juice. Measurements were conducted by enzymatic assay at 340 nm. Arrow shows the stage of female and male flowering. Data points indicate means ± SE (*n* = 3). (**B**) Hexose/Sucrose ratios (hexose/sucrose ratio; means ± SE, *n* = 3) for the stages shown in (**A**).

To evaluate carbohydrate dynamics in the maize stem at different reproductive stages, non-structural carbohydrates were quantified in the stem juice of maize hybrids from 60 DAS (R1 stage) to 102 DAS. Figure 1A shows the carbohydrate mobilization pattern. There is a maximum peak of hexose accumulation at the female and male flowering stages (74–81 DAS). Additionally, an increase in the sucrose level is observed starting at 74 DAS (Figure 1A). Starch, in contrast to sucrose, is a very large and complex molecule. Due to its molecular weight and chemical characteristics, it cannot be moved without degradation, so it accumulates at the location where it is synthesized. The starch level in stem juice was very low at reproductive stages (Figure 1A). A high hexose to sucrose ratio was observed at early reproductive stages but decreased dramatically at 81 DAS (Figure 1B). This could be related to the senescence of the stem after the female inflorescence pollination and the grain filling. Patterns of non-structural sugar behavior and hexose/sucrose ratio were confirmed through the analysis of a second hybrid (Figure S1).

### 3.2. The Female Inflorescence before Pollination Has High Sink Strength

**Figure 2:**
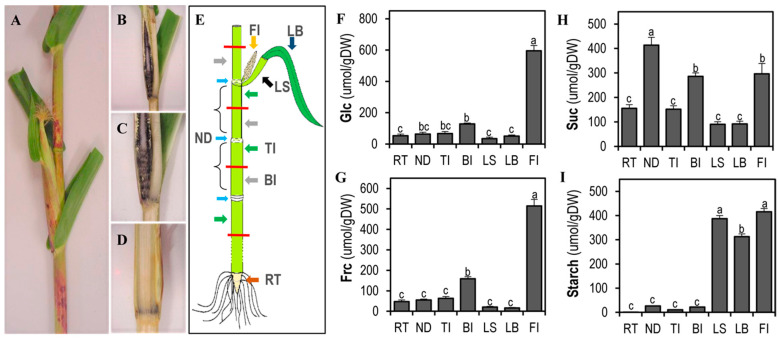
Starch staining and non-structural carbohydrate levels in Puma maize hybrids at 60 DAS (R1 stage). (**A**) Maize stem at 60 DAS showing female inflorescence with emerging stigmas. (**B**–**D**) Longitudinal cuts of the stem and the female inflorescence stained with iodine (KI-I_2_) showing starch accumulation. (**E**) Diagram of different tissues sections collected. (**F**–**I**) Carbohydrate quantification of Puma hybrid tissues at 60 DAS. Plants were divided into different tissue sections: root, RT; stem node, ND; top internode, TI; bottom internode, BI; leaf sheath, LS; leaf blade, LB; and female inflorescence, FI. (**F**) glucose, Glc; (**G**), fructose, Frc; (**H**) sucrose, Suc; and (**I**) starch levels in the different tissues. Bars are the average of *n* = 10, SE; ANOVA, Fisher-LSD test *p* = 0.05; means with the same letter (a, b or c) are not significantly different.

To characterize the starch patterns in the different maize tissues, iodine staining was used. The Puma hybrid at 60 DAS was chosen as our model due to the pattern of carbohydrate accumulation in the stem (Figure 2). At 60 DAS, Puma hybrid plants were at reproductive stage R1, the stem had reached its maximum height and female inflorescences were obvious, with the stigma emerging 2–3 cm outside of the husk leaves (Figure 2A). Longitudinal cuts were made to the stem tissues, and the cuts were stained with iodine to visualize starch accumulation (Figure 2B–D). In Figure 2B,C, a high accumulation of starch in the female inflorescence can be observed, mainly in the peduncle and the border of the corn cob. In Figure 2D, a transversal cut of the stem highlights the accumulation of starch, mainly in the node.

In summary, low levels of starch were observed in the stem (Figure 2). To determine if the same pattern was observed at early stages of stem development, longitudinal and cross sections of 21 DAS maize stems were stained (Figure S2). At this stage, the maize stem is very small (around 6 mm width (Figure S2A,B)), showing high starch accumulation in the shoot tip (Figure S2B,C). Additionally, nodes and internodes can be differentiated due to the starch accumulation. While the nodes were darkly stained (Figure S2C, yellow arrow), the internodes did not stain (Figure S2C, red arrow). In a transverse cut of the node tissues (Figure S2D,E), starch was present in the parenchyma tissues surrounding the bundle sheaths. This suggests that this starch is not synthesized directly from photosynthesis because at this stage, the stem is surrounded by leaf sheaths, and chloroplasts are not present in the middle of the stem. At 21 DAS, the female inflorescence in the Puma hybrids was about 3.6 mm long (Figure S2F–H) and showed high starch accumulation (Figure S2H), while the root did not accumulate starch (Figure S2I,J). Starch accumulation in the stem and the female inflorescence at this stage is consistent with its role as a carbon storage molecule during the development of non-photosynthetic tissues.

Due to the relationship between starch and sucrose in the sink–source mechanism, we quantified carbohydrate accumulation in various tissues of the maize plant. The Puma hybrid plants were divided into stem node, ND; top internode, TI; bottom internode, BI; leaf sheath, LS; leaf blade, LB; female inflorescence, FI; and root, RT (Figure 2E). The carbohydrate levels at reproductive stage R1 are shown in Figure 2F–I. Glucose and fructose can be transported throughout the plant and are often interconverted into more complex carbohydrates. The female inflorescence had a high accumulation of these two hexoses, followed by the bottom internode (Figure 2F,G). Sucrose levels were high in the nodes, followed by the female inflorescence and the bottom internodes (Figure 2H). This makes sense due to the role of this molecule as the main metabolite to be transported to the sink tissues and to the close connection between these tissues. As expected, starch levels were high in photosynthetic tissues such as those in the leaf blade (LB) and leaf sheath (LS) (Figure 2I). The female inflorescence also had high starch levels, but in contrast to leaf tissue, the female inflorescence is a sink tissue. Female inflorescence and leaf starches are different: leaf starches are transitory, while the starch in the female inflorescence is a reserve starch such as the one found in stem tissue. Finally, at 60 DAS, starch levels in the different sections of the stem tissue (ND, TI, BI) were not significantly different from those found in the root tissue (Figure 2I).

To compare carbohydrate composition at vegetative stages with early reproductive stages, vegetative tissues were collected from the Puma hybrid plants at stage V12, before female inflorescences were visible. Glc levels were similar in the vegetative tissues at the V12 stage (ND 62.9 µmol/g; TI 57.5 µmol/g; RT 53.7 µmol/g; LB 42.4 µmol/g; LS 39.0 µmol/g) and in the reproductive tissues at the R1 stage (ND 63.1 µmol/g; TI 67.0 µmol/g; RT 52.87 µmol/g; LB 50.7 µmol/g; and LS 34.4 µmol/g) (Figure 2F–I and Figure S3). Starch levels were also similar between these two stages in most of the tissues, with the exception of the ND, which had 60.5 µmol/g at the V12 stage and 26.7 µmol/g at the R1 stage (Figure 2F–I and Figure S3). The sucrose level nearly doubled from the V12 stage (ND 199.3 µmol/g; TI 86.1 µmol/g; BI 143.2 µmol/g; RT 78.1 µmol/g; LS 53.4 µmol/g) to the R1 stage (ND 413.9 µmol/g; TI 151.6 µmol/g; BI 286.1 µmol/g; RT 155.8 µmol/g; LS 90.6 µmol/g) (Figure 2F–I and Figure S3). The root tissue had similar levels of Frc and Glc, but starch was almost undetectable at both the V12 stage (3.9 µmol/g) and at the R1 stage (1.23 µmol/g). At the V12 stage, the top internode section had Glc (57.5 µmol/g), Suc (86.1 µmol/g), and starch (6.4 µmol/g) patterns that were similar to those of the root tissue (53.7 µmol/g, 48.9 µmol/g, and 3.9 µmol/g, respectively) (Figure S3). Fructose was higher in the bottom internode, but glucose levels were similar in the different tissues (Figure S3). Both hexoses and metabolite levels can change constantly due to their role as part of other complex metabolites such as sucrose and starch. Starch accumulation in the leaves was also high (Figure 2I and Figure S3D), while hexose and sucrose levels were lower than they were in the other tissues (Figure 2F–H and Figure S3A–C). In summary, iodine staining showed similar levels of starch in the female inflorescence and the stem sections at early developmental stages. At reproductive stage R1, differential starch and hexose/sucrose levels in the female inflorescence and stem suggest that contrasting sucrose–starch metabolism occurs in those tissues.

### 3.3. Differential Gene Expression between the Female Inflorescence and the Top Internode of the Stem

**Figure 3:**
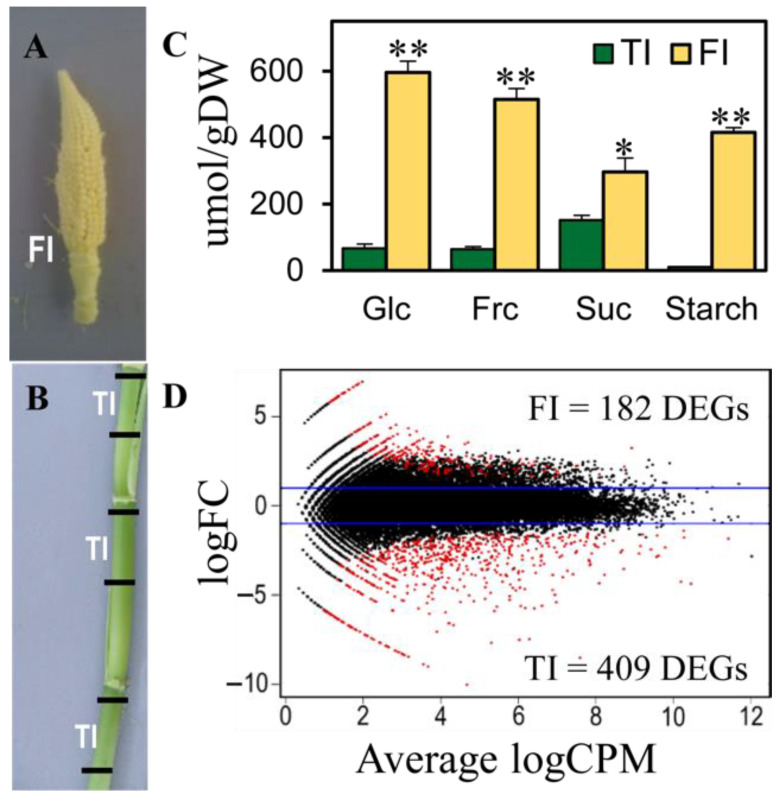
Transcriptomic comparison of the female inflorescence and top internode of the maize stem at 60 DAS. (**A**,**B**) Tissues used to build the libraries for transcriptomic analysis; (**A**) FI, female inflorescence and (**B**) TI, top internode. (**C**) Carbohydrate level differences between FI and TI; mean ± SE, *n* = 10, unpaired *t*-test, df = 10, * *p* < 0.05, ** *p* < 0.01. (**D**) Differentially expressed genes (DEGs) between FI and TI; DEGs visualized as a MA plot (log ratio vs. abundance) where each dot represents a gene, and a red dot represents a significantly DEG where FDR < 0.05 and log_2_FC (log of fold change) > |2|; log_2_CPM, log of count per million.

To explore global gene expression changes between tissues with different carbohydrate levels, transcriptomic analyses of the female inflorescence before pollination (FI, Figure 3A) and the top internode of the maize stem (TI, Figure 3B) were performed at 60 DAS (reproductive stage R1). RNA-seq data from each tissue was mapped to the B73 reference genome (B73 RefGen-v4). A total of 591 genes with an FDR < 0.05 and log_2_FC > |2| were considered as differentially expressed genes (DEGs) (Figure 3D; Table S2). A total of 182 genes were more highly expressed in the female inflorescence (FI) compared to the top internode of the stem (TI), while 409 were more highly expressed in the top internode (TI) of the stem compared to the female inflorescence (FI) (Figure 2D). Of these 591 DEGs, 67 were transcription factors, of which 15 were more expressed in the FI, and 52 were more expressed in the TI (Table S2).

**Figure 4:**
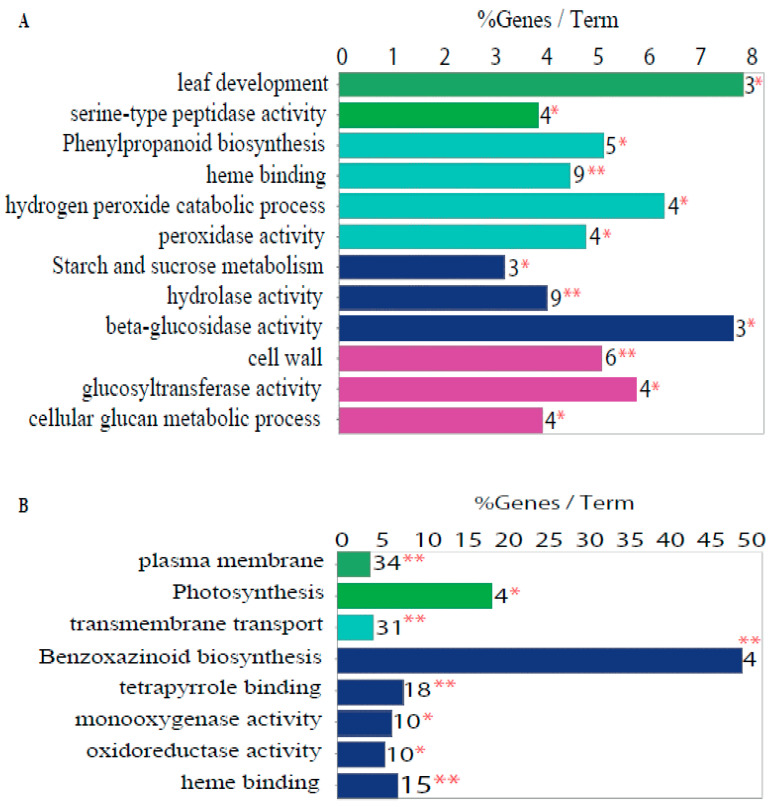
Enriched GO terms among DEGs. (**A**) GO terms enriched among DEGs more highly expressed in the female inflorescence (FI). (**B**) GO terms enriched among DEGs more highly expressed in the top internode of the stem (TI). A two-sided hypergeometric test was used to test for enrichment/depletion, Bonferroni step down correction method, kappa score 0.4. The enrichments show only significant GO terms, *p*-value: * 0.05, ** 0.01.

GO term enrichment analyses were performed on the list of DEGs. ClueGO software found some common GO terms between these two tissues, such as “heme binding” (Figure 4; GO:0020037, Table S3). Panther software identified “cellular nitrogen compound” (GO:0034641) and “tetrapyrrole binding” (GO:0046906) (Table S3). The percentage of genes per enriched GO term was low in the FI tissue compared to the TI tissue, which was probably related to the lower number of DEGs that were overexpressed in FI tissue compared to in the TI tissue (Figure 4A). The “sucrose–starch” metabolism GO term containing *Sus5* (Zm00001d051837, a sucrose synthase related to sucrose degradation) and two putative beta-glucosidases (*Bglu3*, Zm00001d028243; *Bglu1*, Zm00001d048055), was enriched in FI tissue (Figure 4A). The TI-enriched GO terms included “photosynthesis”, “response to light stimulus”, and “transmembrane transport” (Figure 4B), all of which are related to the sucrose–starch metabolism and sink–source mechanisms. Analysis with Panther (Table S3) also showed “sucrose” (GO:005986), “fructose 1,6-biphosphate” (GO:0030388), and “fructose” (GO:0006000) metabolic process enrichment in the TI, which could be related to dynamic sucrose metabolism in this tissue.

### 3.4. qRT-PCR Validation of Gene Expression for Sucrose-Starch Metabolism Enzymes

**Figure 5:**
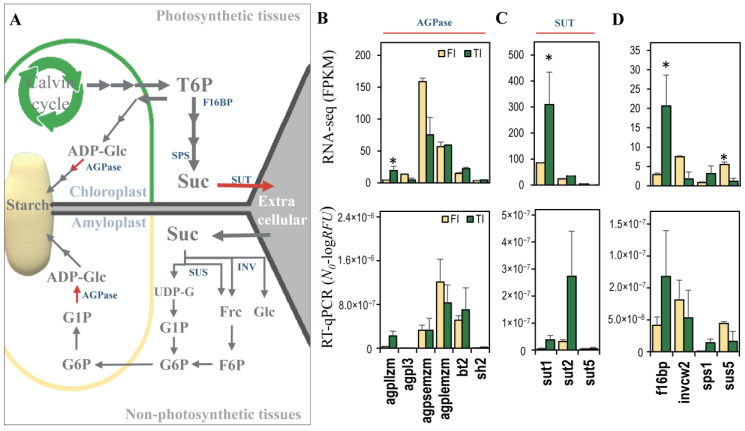
Transcript levels of genes for sucrose–starch metabolism enzymes. (**A**) Summary of sucrose–starch pathway in photosynthetic and non-photosynthetic cells. (**B**–**D**) Transcript quantification by RNA-seq (upper row) and validation by RT-qPCR (lower row). (**B**) ADP-Glucose pyrophosphorilase (AGPase) family, Agpllzm, Agpl3, Agpsemzm, Agplemzm, Bt2, Sh2. (**C**) Sucrose transporter (SUT) family, Sut1, Sut2, Sut5. (**D**) Fructose 1,6 bi-phosphatase (F16bp), Invertase (Invcw2), Sucrose Phosphate Synthase (Sps1), and Sucrose Synthase 5 (Sus5). * Significantly differential expression FDR < 0.05.

Figure 5A shows a summary of key sucrose–starch metabolism enzymes that are active between the photosynthetic and sink tissues. To correlate gene expression with changes in sucrose–starch levels between the female inflorescence (FI) and the top internode of the stem (TI), we looked in our transcriptome data for genes involved in the sucrose–starch metabolism. Quantitative real-time PCR (qPCR) validation of transcript levels of differentially expressed sucrose–starch metabolism genes from Table S2 showed expression patterns that were similar to the RNAseq data (Figure 5B–D). A member of the AGPase family, the *Agpllzm* large subunit (Zm00001d033910), was significantly overexpressed in the RNAseq data in the TI compared to FI tissue, and a similar pattern was seen by qPCR (Figure 5B). Putative sugar transporters were expressed in the top internode of the stem tissue (Figure 5C). Consistent with the differential hexose/sucrose ratio at the R1 stage in the stem, the *Sut1* isoform (Zm00001d027854) was significantly more highly expressed in the RNAseq data in TI compared to FI; this gene also showed higher TI expression in the qPCR experiment (Figure 5C). Among the other enzymes related to sucrose metabolism, RNAseq analysis found a member of the fructose-1,6-biphosphate family (*F16bp*, Zm00001d028562) that was significantly more expressed in TI than in FI, a trend that could also be seen by qPCR validation (Figure 5D). *Sus5* (Zm00001d051837), a member of the sucrose synthase family that participates in sucrose degradation (Figure 5A), was significantly higher in FI than in TI in the RNAseq experiment, a trend that was also seen by qPCR (Figure 5D). Isoforms of other sucrose metabolism enzymes such as a cell wall invertase (*Invcw2*, Zm00001d003776) and a sucrose phosphate synthase (*Sps1*, Zm00001d012036) were also measured; their expression by qPCR was similar to the expression shown by the transcriptome analysis (Figure 5D).

### 3.5. The Expression of the bZip113 and Ereb17 Transcription Factor Genes Was Highly Correlated with the Expression of Sucrose Transporters

**Figure 6:**
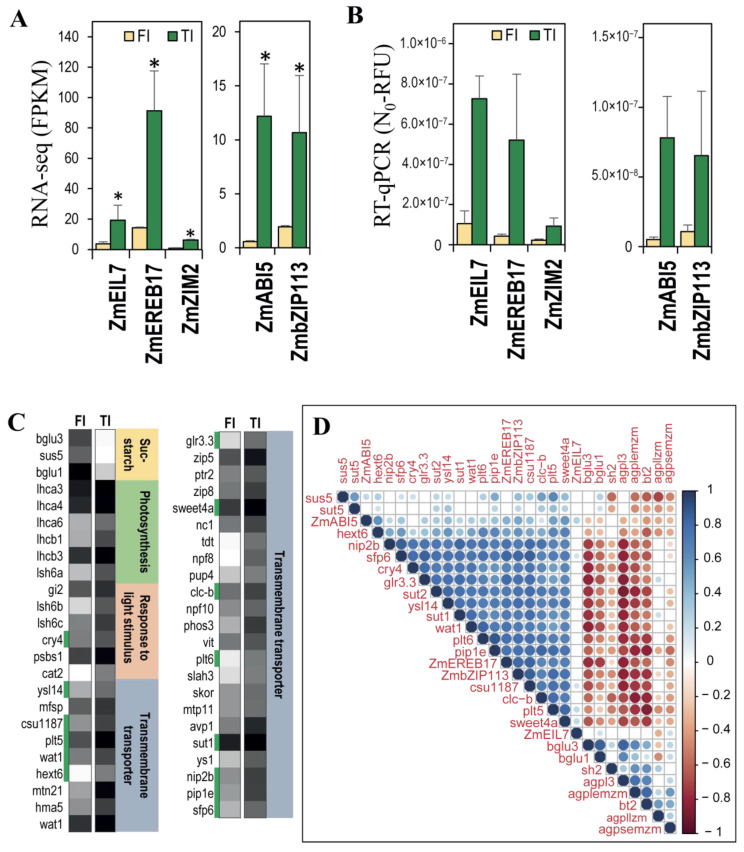
Validation of differentially expressed genes in the female inflorescence vs. top internode section of stem tissue. (**A**) Gene expression of transcription factors by RNA-seq and (**B**) validation by RT-qPCR; * significant differentially expressed, FDR < 0.05. (**C**) Differentially expressed genes from our RNAseq experiment for enriched GO terms related to sucrose–starch metabolism in the female inflorescence (FI) and the top internode (TI); green marks refer to the large cluster formed in the correlation graph in (**D**); FDR < 0.05. (**D**) Expression correlation of differentially expressed transcription factors, AGPase family, sucrose transporter, and transmembrane transporters from the maize B73 genome atlas. Spearman coefficient correlation (SCC), *p*-value < 0.01; colored circles correspond to the significant correlations; non-significant correlations are blank squares.

To explore whether the differential expression of sucrose–starch metabolism enzymes might be correlated with transcription factor expression, five transcription factor genes that were significantly more highly expressed in TI than in FI tissues in the RNAseq experiment were validated by qPCR (Figure 6A). In agreement with the RNAseq results, qPCR analysis showed higher expression of *Eil7* (Zm00001d003451), *Ereb17* (Zm00001d052229), *Zim2* (Zm00001d013331), *Abi5* (Zm00001d013722), and *bZip113* (Zm00001d026398) transcription factors in TI tissue (Figure 6B). To test the correlation between differentially expressed genes from the most enriched GO terms in Figure 4, genes related to sucrose–starch metabolism in each tissue were extracted (Figure 6C). Only three genes related to the “sucrose–starch” GO term were more expressed in the FI tissue (Figure 6C). The most enriched GO term in the TI tissue was “transmembrane transporter”, followed by “photosynthesis” and “response to light stimulus” (Figure 6C). In the “photosynthesis” GO term, we can see some members of the light-harvesting chlorophyll a/b complex family (*Lhca3, Lhca4, Lhca6, Lhcb1, Lhcb3*), which may be expressed in the photosynthetic epidermis of the stem. Sugar transporter genes found in the “transmembrane transporter” GO term include *Hext6* (*Hexose transporter 6*), *Sweet4a* (*Sugars will eventually be exported transporter 4a*), and *Sut1* (*Sucrose transporter 1*).

Figure 6D shows a correlation matrix built with the full transcriptome data from the maize B73 genome atlas. Genes represented here include the differentially expressed transcription factors from Figure 6A,B, sucrose–starch enzymes from Figure 5B–D, and differentially expressed genes from Figure 6C. *Sus5* (*Sucrose synthase 5*) and *Agpllzm* (*ADP-glucose pyrophosphorilase large subunit*) expression were negatively correlated, as observed in the FI and TI transcriptomes (Figure 5B,D). In contrast, a large cluster with a significant positive correlation includes the *Ereb17* and *bZip113* transcription factors, three sucrose transporters (*Sut1, Sut2* and *Sweet4a)*, and many other classes of transporters (green marks in Figure 6C). This suggests that the bZIP113 and ABI51 transcription factors could be involved in the regulation of transport, including sugar transport, in the top internode of the stem. This cluster was negatively correlated with the AGPase isoforms related to starch synthesis (*Agpl3, Agplemzm, Bt2*) as well as *bGlu1* and *bGlu3,* which were more expressed in the FI tissue. Overall, sugar transporter genes showed the highest positive correlation with the *bZip113* and *Ereb17* transcription factors.

## 4. Discussion

### 4.1. Starch Accumulation in Maize Physiology

Insoluble starch is produced from photosynthetically derived sugars and provides plants with a stable and abundant energy source to maintain metabolic needs in the absence of light [12,41]. The exchange of sap containing photosynthetic sugars occurs in the stem nodes between the large phloem vessel and a vascular bundle [42]. Consistent with this, we observed starch accumulation in the parenchyma tissue near the bundle sheath of nodes in the maize shoot tip at early vegetative stages (Figure S2A–E). A similar starch pattern was described in the wheat stem tissue, and the accumulation of starch in the parenchyma tissue has been observed in other plants [43,44]. Starch accumulation could also be observed in the topmost ear shoot at 21 DAS (Figure S2H), suggesting that this starch may be involved in the floral transition, which had already occurred by that time [45]. During vegetative stages, starch in the nodes promotes the growth of young leaves and the elongation of the stem internodes. The localization of starch to the nodes during vegetative developmental stages also likely serves as a reserve for later carbon allocation. We observed residual starch in the nodes and the bottom internodes while the stem was reaching its maximum height (Figure 2D,I and Figure S3D). Comparing the transcriptomes of the female inflorescence and the top internode section of the stem, we found the *Abi5* transcription factor to be more highly expressed in the top internode of the stem compared to the female inflorescence (Figure 6A,B). In Arabidopsis, overexpression of the ABA-insensitive transcription factors *ABI4* and *ABI5* induced effector genes that are involved in seed maturation and reserve storage [46]. *ABI4* is also involved in sugar signaling and the control of photosynthetic and starch biosynthetic genes [47,48].

Unexpectedly, we could not find differentially expressed starch metabolism enzymes between our two transcriptome conditions (Table S2, Figure 5B,C), even when the top internode and the female inflorescence showed different starch levels (Figure 3C). This could be because starch synthesis is no longer active in the female inflorescence at this stage and because the starch present is left over from early stages or because the starch metabolism is regulated at the protein level and does not require differential transcription. However, other GO terms related to starch and sucrose metabolism were enriched in the genes upregulated in the female inflorescence (Figure 4A). The genes belonging to this GO term were members of the SUS family (*Sucrose synthase 5*), which are involved in sucrose degradation, and two putative genes presenting homology to the beta-glucosidase (*bGlu1, bGlu3*) family, which is involved in starch degradation.

At reproductive stage R1, the topmost female inflorescence, leaf sheath, and leaf blade showed high starch accumulation (Figure 2I). Thus, the starch in the stem and in the female inflorescence are synthesized in sink tissues and show a similar synthesis/degradation ratio (Figure S2E and Figure 2B,C). The starch in the female inflorescence is a storage starch that is synthesized in sink tissues, probably in preparation for grain filling, while the starch in the leaves is a transitory starch that is synthesized directly in the photosynthetic tissues [49,50,51,52].

### 4.2. The Function of The Maize Stem in Carbon Partitioning

The stem plays an important role because it connects the source and sink tissues of the maize plant [45]. In the stem juice of both the Puma and Dow2B hybrids, we observed a high rate of sucrose degradation into hexoses from the starting reproductive stage until the pollination stage, after which sucrose started to accumulate, and the hexose–sucrose ratio decreased drastically (Figure 1A,B and Figure S1). Hexose accumulation in the stem may be related to male and female flowering and may precede the start of grain filling. At reproductive stage, the function of the stem seems to be the reallocation of carbon to the tissue with most sink strength, the female inflorescence (Figure 1 and Figure 2F–H). The node tissue becomes relevant as a connection of source and sink tissue, as shown by sucrose accumulation in the stem that is as twice as high as the sucrose accumulation found in the V12 stage (Figure 2H and Figure S3C). By contrast, fructose at the V12 stage is about 1.5 times that of the level of glucose in all of the stem sections (Figure S3A,B), while at reproductive stage, levels of these hexoses are similar. The fact that the nodes function as a carbon partitioning port could be related to the fact that only around 3% of the axial vessels pass through nodes without being interrupted by end walls [42].

Metabolite transport between organelles, cells, and source and sink tissues not only enables pathway coordination, but it also facilitates whole plant communication, particularly in the transmission of information concerning resource availability [53]. The stem functions in the transport of different metabolic substances, as shown by the many transmembrane transporters that were differentially active in stem internode tissue compared to the female inflorescence (Figure 4B and Figure 6C). A correlation analysis built with the transcriptome data of the B73 genome atlas showed that the expression the *bZip113* and the *Ereb17* transcription factors positively correlated with a cluster of these transmembrane transporters (Figure 6D). In this same cluster where we also found sugar transporters, including two sucrose transporters belonging to the SUT family (*Sut1, Sut2*), one from the SWEET family (*Sweet4b*), and a hexose transporter (*Hext*). In some plants, it has been observed that hexoses can be transported through the phloem, a process that is similar to sucrose [54]. The manipulation of sucrose transporters, for example SUT1 and SWEETs, may have a dramatic effect on sucrose remobilization and the source/sink relationships underpinning plant growth and development [53,55,56].

Increasing sink strength potential in the female inflorescence before pollination is vital for the grain filling stage. Once the reproductive stage is reached, the female inflorescence is the principal tissue with high sink strength (Figure 2F–I and Figure 3C). Kernel weight has previously been related to changes in assimilated availability during grain filling, suggesting that maize plants establish an early sink potential [4]. Thus, our results point to the stem as a dynamic reservoir of carbohydrates that may be correspond to the grain filling stage in the female inflorescence.

## 5. Conclusions

Carbon partitioning in the maize stem is a dynamic process that takes place between the vegetative and reproductive stages. Sucrose–starch metabolism plays a prominent role in the sink–source relation during all of the developmental stages. At different reproductive stages, the female inflorescence is the main sink tissue, so reserves accumulate as close to the site of future grain development as possible. The sink–source role of the stem has been studied less. Our results point to a key role for the stem in carbon partitioning and reallocation that is related to the transcription factors *bZip113* and *Ereb7,* which are upregulated in the stem internode tissue and whose expression is correlated with the sugar transporters *Sut1* and *Sweet4a*.

## Data Availability

Raw data have been deposited in the GEO database under accession number GSE181998.

## Supplementary Materials

The following are available online at https://www.mdpi.com/article/10.3390/plants11030238/s1, Figure S1: Carbohydrate quantification in stem juice of Dow2B (yellow grain) hybrid maize at reproductive stages. Figure S2: Starch accumulation in different tissues at 21 DAS. Figure S3: Carbohydrate quantification of Puma hybrid tissues at vegetative stage V12. Table S1: Quantitative real-time PCR primers for sucrose-starch genes and transcription factors. Table S2: Differentially expressed genes between female inflorescence (FI) and top internode section of the stem (TI) maize tissues. Table S3: Extended Panther GO enrichment analysis of the DEGs in the female inflorescence (FI) and top internode of the stem (TI).
