# Supplementary figures and images for: Analysis of Global Gene Expression in Maize (Zea mays) Vegetative and Reproductive Tissues That Differ in Accumulation of Starch and Sucrose
Source: Plants (Basel). 2022 Jan 18;11(3):238. doi: 10.3390/plants11030238 (PMC8838981; doi:10.3390/plants11030238)

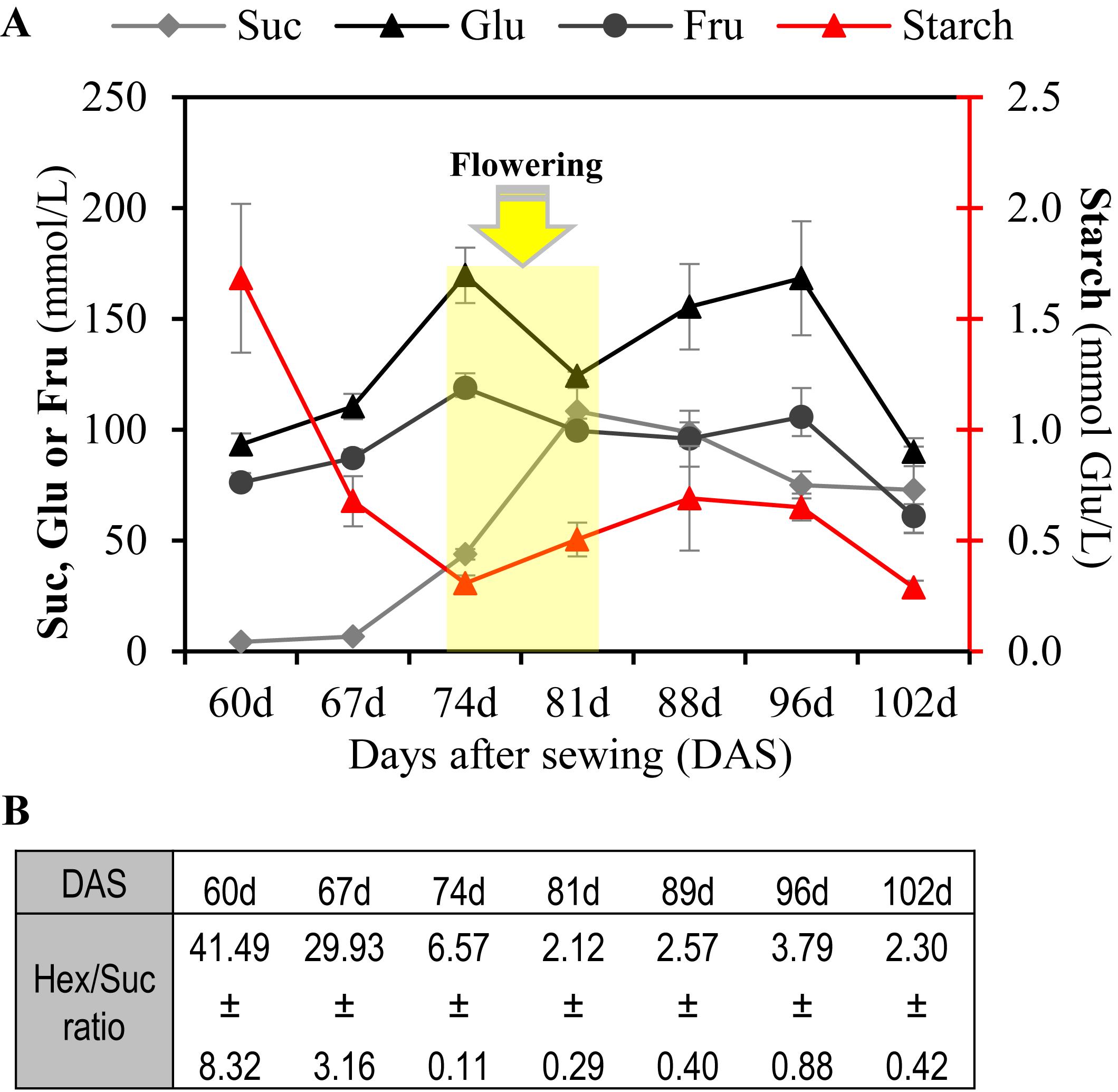

Supplement: Supplementary file 1 [file plants-11-00238-s001.zip › plants-1401381-Figure S1. Carbohydrate quantification in stem juice of Dow2B (yellow.jpg]

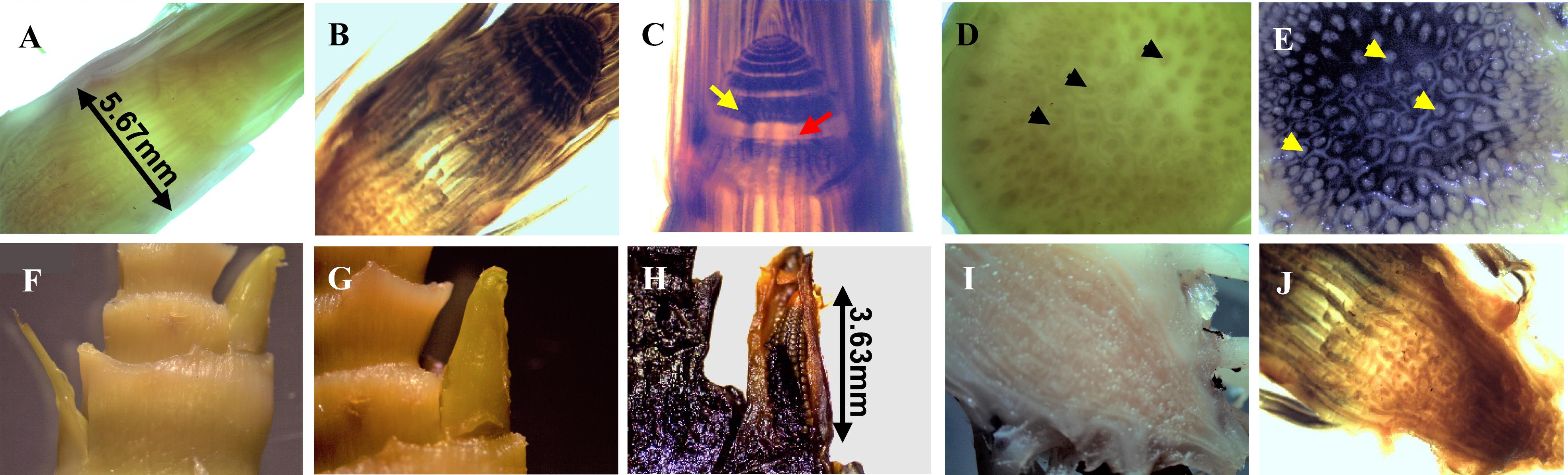

Supplement: Supplementary file 1 [file plants-11-00238-s001.zip › plants-1401381-Figure S2. Starch accumulation in different tissues at.jpg]

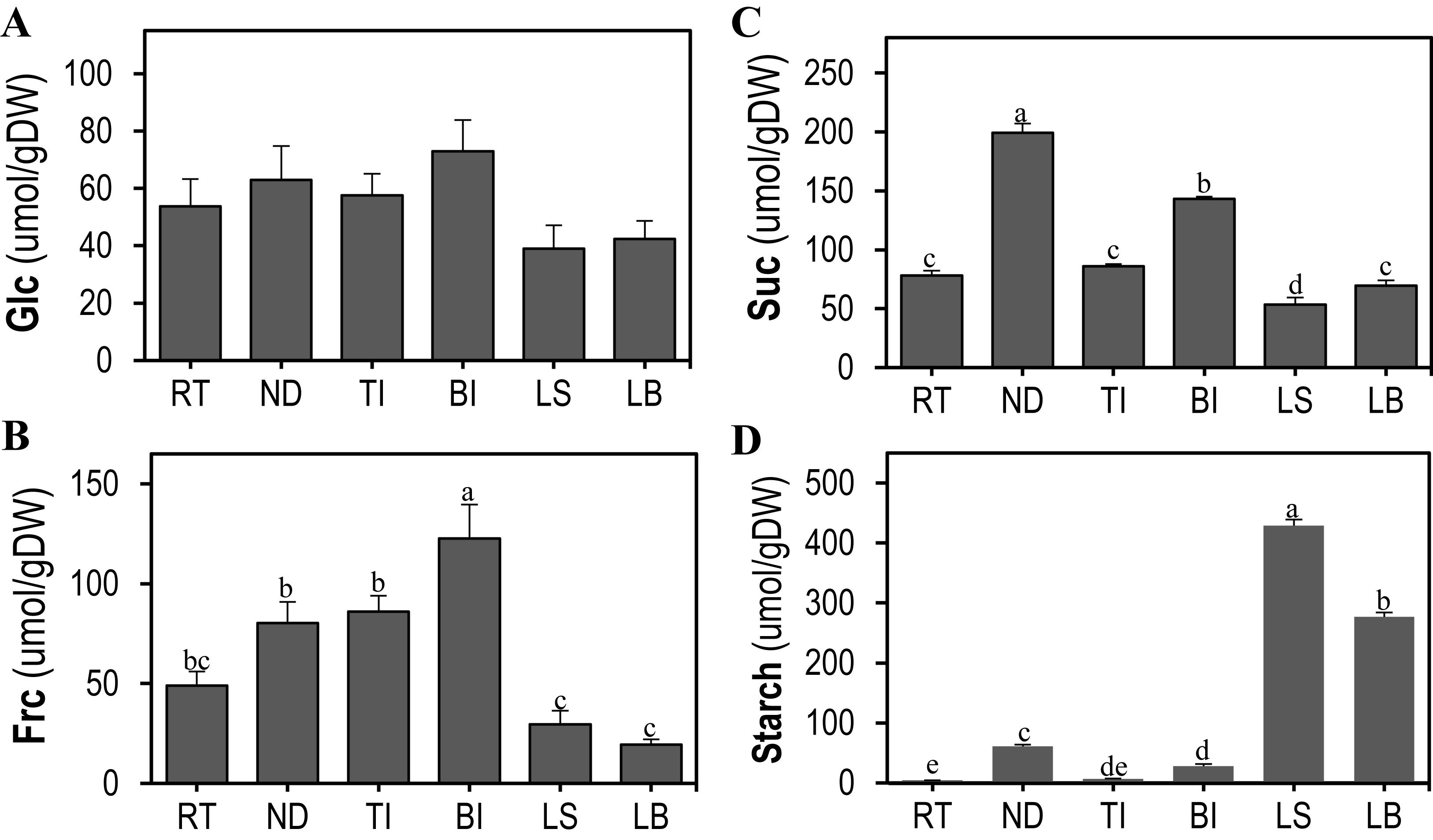

Supplement: Supplementary file 1 [file plants-11-00238-s001.zip › plants-1401381-Figure S3. Carbohydrate quantification of Puma hybrid tissues at vegetative stage V12.jpg]
